# High chromosomal instability in workers occupationally exposed to solvents and paint removers

**DOI:** 10.1186/s13039-016-0256-6

**Published:** 2016-06-20

**Authors:** Mónica Villalba-Campos, Lilian Chuaire-Noack, Magda Carolina Sánchez-Corredor, Milena Rondón-Lagos

**Affiliations:** Facultad de Ciencias Naturales y Matemáticas, Universidad del Rosario, Bogotá, DC Colombia; Department of Medical Sciences, University of Turin, Via Santena 7, 10126 Turin, Italy

**Keywords:** Chromosomal alterations, Chromosomal instability, Occupational exposure, Aromatic hydrocarbons, Comet assay

## Abstract

**Background:**

Painters are exposed to an extensive variety of harmful substances like aromatic hydrocarbons used as solvents and paint removers, some of which have shown clastogenic activity. These substances constitute a complex mixture of chemicals which contain well-known genotoxicants, such as Benzene, Toluene and Xylene. Thus, chronic occupational exposure to such substances may be considered to possess genotoxic risk. In Colombia the information available around the genotoxic damage (Chromosomal and DNA damage) in car paint shop workers is limited and the knowledge of this damage could contribute not only to a better understanding of the carcinogenic effect of this kind of substances but also could be used as biomarkers of occupational exposure to genotoxic agents.

**Results:**

In this study, the genotoxic effect of aromatic hydrocarbons was assessed in peripheral blood lymphocytes of 24 workers occupationally exposed and 24 unexposed donors, by using Cytogenetic analysis and comet assay. A high frequency of Chromosomal alterations was found in the exposed group in comparison with those observed in the unexposed group. Among the total of CAs observed in the exposed group, fragilities were most frequently found (100 %), followed by chromosomal breaks (58 %), structural (41.2 %) and numerical chromosomal alterations (21 %). Numerical chromosomal alterations, fragilities and chromosomal breaks showed significant differences between exposed and unexposed groups. Among the fragilities, fra(9)(q12) was the most frequently observed. DNA damage index was also significantly higher in the exposed group compared to the unexposed group (*p* < 0.000).

**Conclusions:**

Our results revealed that occupational exposure to aromatic hydrocarbons is significantly associated with Chromosomal and DNA damage in car paint shops workers and are also indicative of high chromosomal instability. The high frequency of both Chromosomal Alterations and DNA Damage Index observed in this study indicates an urgent need of intervention not only to prevent the increased risk of developing cancer but also to the application of strict health control and motivation to the use of appropriate protecting devices during work.

## Background

Occupational aromatic hydrocarbons exposure (Benzene, Toluene and Xylene - BTX), mainly via inhalation, occurs most frequently as result of various activities in which these substances are processed, generated or used [1]. These are present in both evaporative and combustive automobile emissions, in cigarette smoke and are commonly used as an industrial solvent in the workplace. Recently, the International Agency for Research on Cancer 2010 (IARC) [2], included in group 1 carcinogens, some aromatic hydrocarbons used as solvents and paint removers, like Benzene, Toluene and Xylene.

Benzene, is a chemical potentially carcinogenic (leukaemogenic) in humans due to the ultimate carcinogen hydroquinone and 1,4-benzoquinone metabolised by cytochrome P450 enzymes (CYP2E1) in the liver [3–6]. Toluene, the methyl- substituted derivative of benzene, is also metabolized by CYP450 enzymes [7]. Although the main products of the benzene metabolism (acid S-phenylmercapturic and trans -trans -muconic) and toluene (hippuric acid) are eliminated through the urine, some intermediate metabolites may generate reactive oxygen species (ROS) and can cause oxidative stress and genetic damage [8]. This genetic damage is mainly represented by DNA adduct formation and impairment of DNA repair mechanisms, DNA single-strand breaks, sister-chromatid exchanges (SCE), micronuclei (MN), DNA cross-linking and Chromosomal Alterations (CAs) in peripheral blood lymphocytes (PBLs) [9–14]. In addition, have been reported that benzene can cause an increased risk of developing cancer in various organs, such as lung, bladder, pancreas and lymphatic and hematopoietic tumors, being it cataloged as clastogenic to human PBLs [10, 15–17].

CAs in PBLs reflect inter-individual sensitivity to exogenous and endogenous genotoxic substances and can be used as biomarkers of genotoxic damage and carcinogenic risk [17–20]. In fact, according to recent reports, CAs represent one of the best internationally standardized and validated biomarkers of early biological effects in human biomonitoring [18, 21, 22]. However, the type and frequency of such CAs have not been carefully characterized in a Colombian population and these could be used as markers of cytogenotoxic damage.

Additional methods are also used in human biomonitoring studies, as the comet assay (gel electrophoresis of individual cells). The comet assay, has proved its usefulness and versatility in human biomonitoring, ecogenotoxicology, genotoxicity testing and basic research into the mechanisms of DNA damage and repair [23–25]. It detects strand breaks and alkali-labile sites at frequencies from a few hundred to several thousand breaks per cell. The use of this assay have greatly increased during the past few decades [26, 27] and allowed evaluating the DNA damage index (DNA-DI) caused by occupational exposure to chemicals.

The aim of the present study was to evaluate the genotoxic effect of occupational exposure to aromatic hydrocarbons used as solvents and paint removers, on the frequency of CAs and on the DNA-DI in PBLs of workers at ten car paint shops at the “7 de agosto” neighborhood in Bogota DC, Colombia.

## Methods

### Study population

The study was carried out in a group of 24 men selected at random and routinely “exposed” to solved paints, at ten car paint shops at the “7 de agosto” neighborhood in Bogota DC, Colombia. The exposed donors consisted of men between 21 and 73 years old and a work time exposed to organic solvents of at least 1 year. Individuals who had suffered from hepatitis or cancer, or had been under chemotherapy or radiotherapy or any other recent prolonged medical treatment were excluded of this study.

The unexposed group was recruited in another area within the same neighborhood, with similar characteristics except for the presence of nearby car paint shops. This group consisted of 24 healthy men, without indication of previous occupational exposure to aromatic hydrocarbons and whose ages were similar to the exposed donors (between 20 and 72 years old) (Table 1).

**Table 1:** Characteristics of the Study population

	Exposed			Unexposed		
Characteristic	N	Mean ± SD/%	Range	N	Mean ± SD/%	Range
Age (years)	24	44.25 ± 13.42	21–73	24	44.45 ± 12.91	20–72
Exposure time (years)	24	18.4 ± 11.1	1–35	24	0	0
Smoking habits						
Smokers	3	12.5		3	12.5	
No smokers	21	87.5		21	87.5	
Alcohol consumption						
Drinkers	21	87.5		17	70.9	
No drinkers	3	12.5		7	29.1	

*SD* Standard Deviation

Data from the 24 exposed donors were compared with those of the unexposed donors. Each donor was personally interviewed by filling in a routine questionnaire including age, smoking and drinking habits, exposure to organic solvents, diseases, occupational history including duration of exposure to chemicals and the use of protecting devices during work (Table 1).

### Blood sampling

Two peripheral blood samples were collected from exposed and unexposed donors by venipuncture. One blood sample was used for Cytogenetic analysis and the other for lymphocytes isolation and further comet assay. The samples were labeled, transported to the laboratory and processed within 3 to 4 h.

### Metaphase spreads and high-resolution GTG

Metaphases were obtained using standardized harvesting protocols for conventional cytogenetic analysis. Briefly, 0.8 ml of heparinised peripheral blood were cultured in duplicates in 10 ml RPMI-1640 medium (Sigma, St. Louis, MO, USA), supplemented with 10 % fetal bovine serum (FBS) (Sigma) and 0.5 % phytohemaglutinin-M (Gibco, Life Technologies, Nebraska, USA). The cultures were incubated at 37 °C and 5 % CO_2_ for 72 h. After this, colcemid solution (N-Deacetyl-N-methylcolchicine, 0.0001 g/ml final conc.) (Sigma) was added to cultures 25 min before cell harvesting [28]. Cells were then treated with hypotonic solution (KCl 0.075 M), fixed three times with Carnoy’s fixative (3:1 methanol to acetic acid) and spread on glass. Glass slides were baked at 80 °C for 2 h, incubated in 2xSSC buffer, placed in trypsin solution (0,25 %) (Gibco) before treatment with Giemsa stain (Sigma).

### Chromosome analysis

Characterisation of CAs was performed in a total of 1018 metaphases. Image acquisition and subsequent karyotyping of metaphases was performed using a Nikon microscope (Applied Imaging, Santa Clara, CA, USA). Numerical (NCAs) and structural chromosomal alterations (SCAs), Fragilities (FRA), chromosome breaks (chrb) and chromatid breaks (chrtb), present in at least two or three metaphases were evaluated. All CAs were described according to the International System for Human Cytogenetic Nomenclature (ISCN) 2013 [29].

### Lymphocyte separation

To perform the comet assay the lymphocytes were isolated by Ficoll-1077 (Sigma Aldrich, St. Louis, MO, USA) density gradient centrifugation and washed in phosphate buffered saline (PBS 1X) (Gibco, Life Technologies, Nebraska, USA). The viability of the cells was tested by trypan blue test (Life Technologies, Nebraska, USA) and was kept greater than 90 %. The volume of cell suspension used in the test was 4×10^3^ lymphocytes.

### Comet assay

We used the standard procedure of alkaline comet assay described by Collins et al 2001 [30] using the Trevigen Comet Assay Kit (Trevigen, Gaithersburg, USA).

In order to calculate DNA damage, 100 cells per individual was analyzed by using fluorescence microscope (Nikon Instruments Inc, USA), with a magnification of 100X. All assays were performed in duplicate. The comets were classified through the Comet Score publisher program, in five categories according to the percentage of DNA in the tail, as follows: 0: No damage (<5 %), 1: Low damage (6–25 %), 2: moderate damage (26–50 %), 3: high damage (51–75 %) and 4: severe damage (>76 %) [30–32]. DNA-DI was then calculated according to the formula proposed by Collins et al 2004 [27]: DI = 0(n) + 1(n) + 2(n) + 3(n) + 4(n), where “n” indicates number of cells in each class. Therefore, a DNA-DI could range from 0 (all cells with no tail, 100 cells × 0) to 400 (all cells with maximally long tails, 100 cells × 4).

### Data analysis

All statistical analyses were performed using IBM-SPSS Statistics Developer (Version 21.0 IBM Company, Chicago, IL). Normality of the data was evaluated by Shapiro-Wilk test. Fisher’s exact test and Wilcoxon test were performed to compare the cytogenetic data with parametric a non-parametric distribution, respectively. The comet assay data, which was normally distributed, were analyzed using Student’s *T*-test. *P* values less than 0.05 were considered significant (*p ≤ 0.05 **p ≤ 0.01).

## Results

### Characteristics of study groups

The effect of occupational exposure to aromatic hydrocarbons on DNA in car paint workers and unexposed donors was assessed by Conventional Cytogenetics and the comet assay.

Detailed information of researched groups is displayed in the Table 1. For the exposed group the median time of exposure to aromatic hydrocarbons was 18.4 years and their median age was 44.25 years. A low prevalence of smoking (12.5 %) and a high consumption of alcoholic beverage (87.5 %) were reported in this group. Also a low prevalence of smoking (12.5 %) and a high consumption of alcoholic beverage (70.9 %) were reported in the unexposed group. The results were expressed as mean ± standard deviation (SD).

### Cytogenetic data

**Fig. 1:**
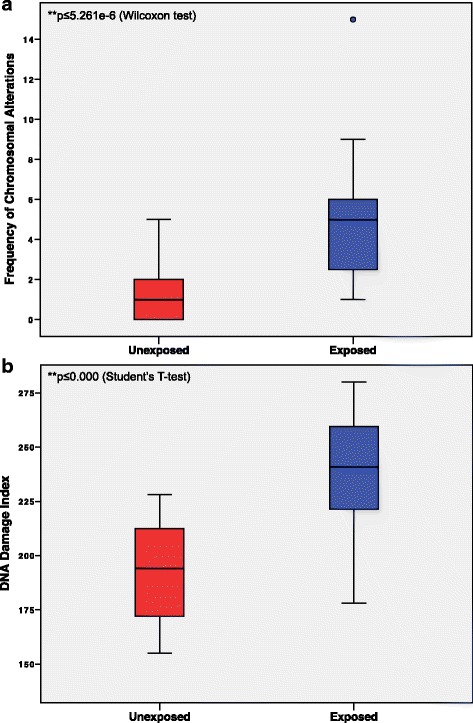
Boxplot of the genotoxic damage observed in the exposed and unexposed groups. **a** Frequency of CAs. The single point at the top of the exposed boxplot represents the maximum number of CAs within this group. **b** DNA damage index (DNA DI). The black line represents the CAs median values. The bottom and top of the boxes represent the 25th and 75th percentile respectively, whereas the box represents the interquartile range

In order to define CAs (Numerical and structural chromosomal alterations, Fragilities, chromosome breaks and chromatid breaks), between 14 and 26 metaphases with good chromosome dispersion and morphology were analyzed for both exposed and unexposed groups. Cytogenetic analysis for both groups demonstrated a modal number diploid (2n). As shown in Table 2 and Fig. 1a, a significant high frequency of CAs was found in the exposed group (119 CAs) in comparison with those observed in the unexposed group (33 CAs) (*p* ≤ 5.261e-06)

**Table 2:** Total number of CAs (by donor) found in the exposed and unexposed groups

No	Exposed	Unexposed	*p*
1	8	5	
2	1	2	
3	7	0	
4	6	0	
5	9	4	
6	15	3	
7	3	4	
8	4	1	
9	6	1	
10	5	1	
11	2	0	
12	6	2	
13	1	2	
14	6	1	
15	8	0	
16	6	1	
17	3	0	
18	2	0	
19	2	2	
20	4	0	
21	5	0	
22	2	2	
23	3	2	
24	5	0	
Total	119	33	5.261e-06^**^
Mean	4.95	1.37	
SD	3.12	1.46	

^**^Difference significant relative to unexposed group at *p* ≤ 0.01 (Wilcoxon test); *SD* Standard Deviation

**Fig. 2:**
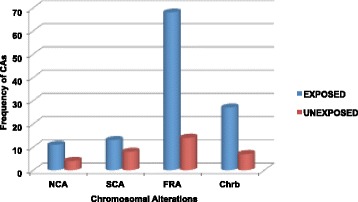
Frequencies of CAs in the PBLs of exposed and unexposed groups. NCAs: Numerical chromosomal alterations; SCAs: Structural chromosomal alterations; FRA: Fragilities: Chrb: Chromosomal breaks

**Fig. 3:**
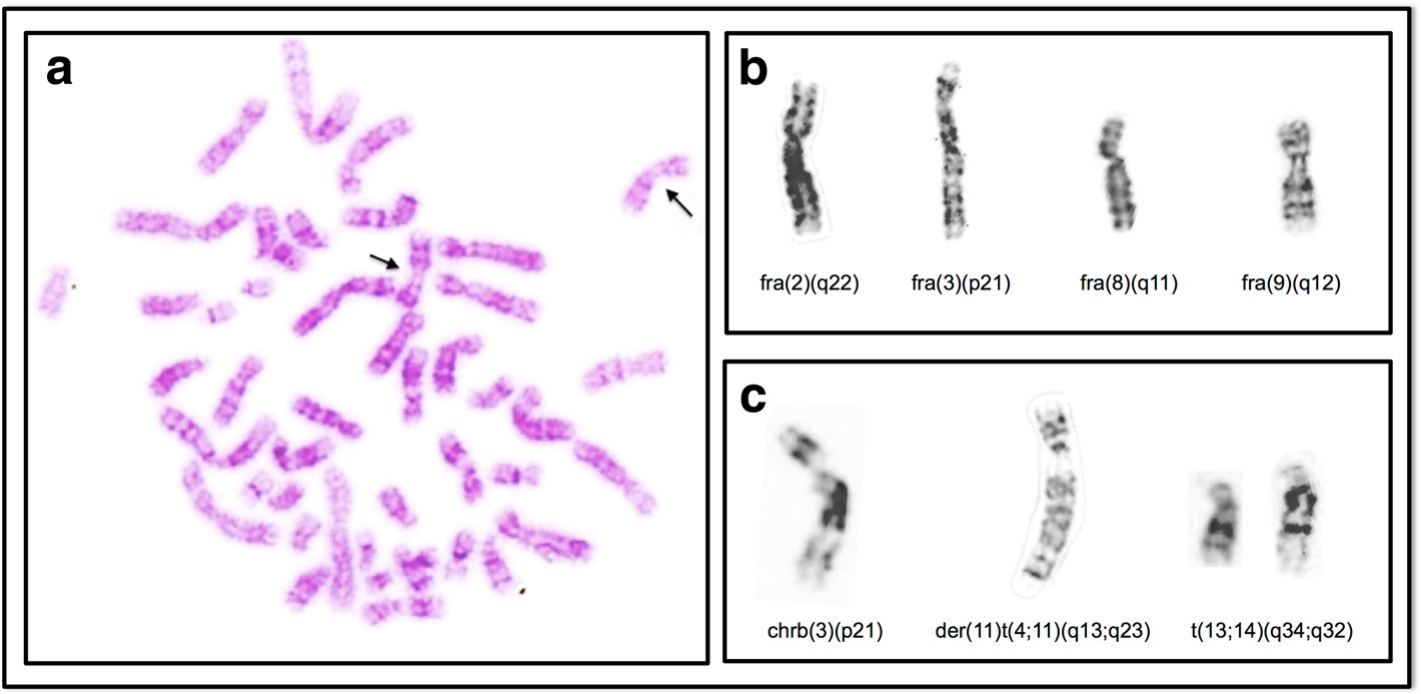
Representative images of CAs observed in the exposed group. **a** G-banding metaphase showing fragility of both chromosomes 9 (fra(9)(q12) (*indicated by arrows*). **b** Chromosomes showing fragilities found in higher frequency. **c** Chromosomes showing chrb and chtb and SCAs

Among the total of CAs observed in the exposed group, Fragilities (FRA) were most frequently found (68/119) (Table 3, Figs. 2, 3a and b), followed by chromosome breaks (chrb) (22/119) and chromatid breaks (chtb) (5/119) (Fig. 3c), structural chromosomal alterations (SCAs) (13/119) (Fig. 3c) and numerical chromosomal alterations (NCAs) (11/119) (Fig. 2). NCAs, FRA, chrb and chtb showed significant differences when exposed and unexposed groups were compared (*p* ≤ 0.01, *p* ≤ 0.000001 and *p* ≤ 0.017 respectively) (Table 3). No significant differences were found in the number of CAs between smokers and nonsmokers.

**Table 3:** Percentage of chromosomal alterations identify in the exposed and unexposed groups

Chromosomal alterations	Exposed %	Unexposed %	*p*
NCAs	21	8.3	0.01^**^
SCAs	29	16.6	0.06
FRA	100	37.5	0.0001^**^
chrb	37.5	16.6	0.0023^**^
chtb	21	4.1	0.0004^**^

*NCAs* Numerical chromosomal alterations, *SCAs* Structural chromosomal alterations, *FRA* Fragilities, *chrb* Chromosomal breaks, *chtb* Chromatid breaks

^**^Statistically significant difference relative to unexposed group at *p* ≤ 0.01 (Fisher’s exact test)

#### Structural and numerical chromosomal alterations

A higher frequency of NCAs and SCAs was identified in the exposed group (11 and 13 respectively) compared to those observed in the unexposed group (4 and 8 respectively). However SCAs alterations were observed only in individual cases and no statistically significant differences were found in the number of donors affected by them (Table 3).

The NCAs identify in both groups were mainly represented by monosomies, whereas trisomies were absent. In the exposed group were found monosomies of the chromosomes 6, 8, 18, 19 and 21, while in the unexposed group were observed monosomies of chromosomes Y and 22.

In the exposed group were observed 13 SCAs in seven donors, including deletions (del), duplications (dup), translocations (t and der), additional material of unknown origin (add) and isochromosomes (i) (Table 4). Among the total of SCAs, deletions were most frequently found (38.5 %), followed by translocations (30 %) and duplications (15 %) (Fig. 3c). In the unexposed group were observed 8 SCAs (deletions and isochromosomes) in four donors (Table 4).

**Table 4:** Structural chromosomal alterations observed in the exposed and unexposed groups

Number	Structural alterations	
	Exposed	Unexposed
1	del(Y)(q12)	del(2)(q16)
2	del(2)(q32)	del(6)(q22)
3	?i(5)(q10)	add(7)(p22)
4	del(5)(q11.2)	inv(7)(p21q36)
5	dup(6)(p22p23)	del(9)(p21)
6	der(7)t(2;7)(q32;q36)	del(10)(p12)
7	der(7)t(7;14)(q32;q22)	del(10)(p13)
8	add(11)(p15)	i(11)(q10)
9	der(11)t(4;11)(q13;q23)	
10	t(13;14)(q34;q32)	
11	del(14)(q22)	
12	del(17)(q24)	
13	?dup(17)(q22q25)	

#### Fragilities

A high frequency of FRA was found in the exposed group (68 fragilities) which showed a statistically significant difference (*p* ≤ 0.0001) as compared to unexposed group (14 fragilities) (Table 3). In the exposed group, the chromosomes X, 1, 2, 3, 5, 6, 8, 9, 10 and 12 showed higher number of fragilities, being the fra(9)(q12) the most frequent (29/68) and present in the 75 % of the exposed (*p* ≤ 0.0001) (Table 5, Fig. 3a and b), followed by fra(1p) and fra(8q) (Table 5). In the unexposed group the fra(9)(q12) was also the most frequent (5/14) in the 16.6 % of the donors.

**Table 5:** Fragilities more frequently observed in the exposed and unexposed groups

Fragilities	Exposed %	Unexposed %	*p*
fra(1)(p)	21	4	0.0004**
fra(2)(q)	16.6	8	0.08
fra(3)(p)	21	12.5	0.18
fra(8)(q)	21	0	0.0001**
fra(9)(q12)	75	16.6	0.0001**

**Statistically significant difference relative to unexposed group at *p* ≤ 0.01 (Fisher’s exact test)

#### Chromosome and chromatid breaks

In addition to fragilities, a higher frequency of chrb and chtb were also observed in the exposed group (22 and 5 breaks respectively) compared to those observed in the unexposed group (6 and 1 breaks respectively). Chromosomes most affected by such changes in the exposed group were chromosomes 1, 3, 6 and 9 (Table 6, Fig. 3c). Comparison of presence of chrb and chtb between exposed and unexposed donors showed a significant difference (*p* ≤ 0.0023 and *p* ≤ 0.0004 respectively) (Table 3).

**Table 6:** Chromosomal breaks (chrb) (A) and chromatid breaks (chtb) (B) more frequently observed in the exposed and unexposed groups

A)			
chrb	Exposed %	Unexposed %	*p*
chrb(6)(q11)	16.6	0	0.0001**
chrb(9)(q12)	12.5	0	0.0002**
B)			
chtb(1)(p11)	12.5	4	0.03*
chtb(3)(p12)	8	0	0.006**

*Statistically significant difference relative to unexposed group at *p* ≤ 0.05 (Fisher’s exact test)

**Statistically significant difference relative to unexposed group at p ≤ 0.01 (Fisher’s exact test)

### Comet assay

The comet assay data for exposed and unexposed groups are presented in Table 7 and Fig. 1b. The DNA damage index (DNA-DI) observed in the exposed group was significantly higher than that observed in the unexposed group (235.7 ± 29.2 and 193.7 ± 22.2, *p* < 0.000, respectively). Smoking habits had no significant effect on DNA-DI among the exposed and unexposed groups.

**Table 7:** Evaluation of DNA damage index (DNA-DI) through Comet assay

Groups	DNA-DI
Exposed (*n* = 24)	235.70 ± 29.23**
Smokers (*n* = 3)	211.66 ± 31.56
No Smokers (*n* = 21)	239.14 ± 28.00
Alcohol Drinkers (*n* = 21)	231.85 ± 29.19
No Alcohol Drinkers (*n* = 3)	262.66 ± 7.50
Unexposed (*n* = 24)	193.79 ± 22.22
Smokers (*n* = 3)	197.33 ± 16.66
No Smokers (*n* = 21)	193.28 ± 23.23
Alcohol Drinkers (*n* = 17)	193.88 ± 25.25
No Alcohol Drinkers (*n* = 7)	193.57 ± 13.89

The values are expressed as mean ± SD. **Statistically significant difference relative to unexposed group. *p* ≤ 0.000 (Student’s *t*-test)

## Discussion

The aim of the present study was to evaluate the genotoxic effect of occupational exposure to aromatic hydrocarbons used as solvents and paint removers, on the frequency of CAs and on the DNA-DI in PBLs of workers at ten car paint shops at the “7 de agosto” neighborhood in Bogota DC, Colombia.

Car paints shop workers are occupationally exposed to a wide range of aromatic hydrocarbons including BTX. Exposure to aromatic hydrocarbons, may take place via vapor inhalation or absorption through the intact skin. Many epidemiological studies have showed a clear relationship between the increase genotoxic damage and exposure to aromatic hydrocarbons, being neccesary to provide more relevant information regarding to the possible deleterious damage upon chromosomes. Although many studies have been conducted around of this topic, the specific type and frequency of such CAs have not been carefully characterized and these could be used as biomarkers of genotoxic damage and as predictors of future cancer risk. To the above is added that in Colombia the information available around the cytogenotoxic damage in car paint shop workers is limited.

The results obtained using high-resolution G-Banding analyses of a large number of metaphases, allowed us to detect previously unreported CAs in individuals exposed to BTX. The mean of CAs found in the exposed group was 3.6 times higher than in the unexposed group, thus indicating a potential cytogenetic hazard due to exposure to aromatic hydrocarbons used as solvents and paint removers in car paint shops.

Among the chromosomal alterations observed at high frequency in the exposed group, monosomies, fragilities, chbr and chrb showed statistically significant differences when were compared to those observed in the unexposed group. In several recent studies, this type of chromosomal alterations have been correlated with a heightened risk of cancer, especially hematological malignancies [33, 34]. For instance, monosomies, observed in gas station attendants and in individuals occupationally exposed to formaldehyde [35, 36], were associated with myeloid malignancies [37]. Further, monosomies of chromosomes 18 and 19, observed by us in the exposed group, were associated with risk of developing leukemia in individuals occupationally exposed to benzene [33]. Nevertheless, although the structural chromosomal alterations identify in the exposed group were not significant, some of them, including del(5)(q) and t(11)(q23), were detected in leukemia patients with likely prior exposure to benzene [12, 38].

In addition, a significant increase in the frequency of FRA, chrb and chtb in exposed compared to unexposed group was also observed. FRA, chrb and chtb are unstable CAs and regions of potential genome instability [39] that can lead to the formation of cancer-specific CAs such as translocations, deletions [39–43], duplications, amplifications [44, 45], sister chromatid exchanges [46], intrachromosomal gene amplification [47] and other chromosomal changes associated with human diseases [48, 49]. Further, many genes identified as tumor suppressors or oncogenes are located at or within fragile sites [50].

Among the FRA identified in this study, fra(9q12) was the most frequently observed in the exposed group. High frequency of this fragility was previously reported by us in a Colombian population with breast cancer [51]. On chromosome 9 are located the tumor suppressor genes *CDKN2A*, *PIPSK1B, BTEB1, RECK* and *BAG1*, the latter associated with antiapoptotic functions and overexpressed in invasive breast carcinomas.

Given the significantly high frequency of **fra(9)(q12)** in the exposed group, as well as its previous observation in patients with breast cancer, we considered that this fragility could be postulated as a cytogenetic biomarker of genotoxic damage associated to occupational exposure to BTX.

Our findings are consistent with previous reports in which increased chromosomal abnormalities in PBLs were associated with occupational exposure to benzene [3, 9, 35, 36]. However although most studies have mostly shown positive results, others have not found any association [52, 53].

Additionally, it is important to emphasize that increased genotoxic damage and, therefore a higher risk to develop cancer, have also been associated with other specific job occupations. For instance, a higher risk to develop hematologic diseases was observed in benzene exposed oil refinery workers [9], workers exposed to low levels of formaldehyde [35, 54], gas station attendants [36], painters [55] and petroleum refinery workers [56]. Further, an increased risk of laryngeal cancer was also reported in production-related workers, transport equipment operators, miners, tailors, blacksmith and toolmakers, painters, bricklayers and carpenters [57].

In our study, there was a significant increase in DNA-DI, evidenced by the comet assay, in exposed in comparison to the unexposed group. Comet assay has been used as a sensitive biomarker that reveals DNA damage caused either directly by reactive oxidant agents, or indirectly by substances that can generate free radicals [58, 59]. These findings are consistent with previous studies [55, 56, 60, 61] and allow us to confirm that occupational exposure to BTX induce genotoxic damage at both DNA and chromosomal level. However, we also note that smoking habit had no significant effect on DNA-DI among exposed and unexposed groups, which could be due to low cigarette consumption among the workers (1–3 cigarettes per day). Lack of association between DNA-DI and smoking habit in PBLs of individuals occupationally exposed to aromatic hydrocarbons, have been also indicated by several studies [55, 62–67].

In summary, our results demonstrated that occupational exposure to BTX is significantly associated with chromosomal and DNA damage in car paint shops workers and are indicative of high chromosomal instability (CIN). CIN, defined as a state of continuous formation of novel chromosome mutations at a rate higher than in normal cells, could predispose cells to further mutations and by that to an increased risk of malignant transformation [68, 69]. In fact, several prospective cancer studies have shown a linear trend between CAs in PBLs and subsequent cancer risk [35–37, 54, 70–73].

## Conclusions

BTX occupational exposure of car paints workers represents a relevant risk factor for the development of diseases associated with genetic damage. The high frequency of CAs and the high DNA-DI observed in this study indicate an urgent need of intervention not only to prevent the increased risk of developing cancer but also the application of strict health control and motivation to the use of appropriate protecting devices during work.

## Abbreviations

BTX, benzene, toluene and xylene; CAs, chromosomal alterations; chrtb/chrb, chromosome breaks and chromatid breaks; CIN, chromosomal instability; CYP450, cytochrome P450 enzymes; DNA-ID, DNA damage index; FRA, fragilities; IARC, international agency for research on cancer; MN, micronuclei; NCAs, numerical; PBLs, peripheral blood lymphocytes; ROS, reactive oxygen species; SCAs, Structural chromosomal alterations; SCE, sister-chromatid exchanges; SD, standard deviation
